# Therapeutic inertia in proteinuria management among type 2 diabetes (T2DM) patients in primary care settings: prevalence and associated risk factors

**DOI:** 10.1186/s12875-021-01455-3

**Published:** 2021-06-21

**Authors:** FU Leung Chan, Yim Chu Li, Xiao Rui Catherine Chen

**Affiliations:** Department of Family Medicine and General Out-Patient Clinics, Kowloon Central Cluster, Hospital Authority, Rm 807, Block S, Queen Elizabeth Hospital, 30 Gascoigne Road, Kowloon, Hong Kong

**Keywords:** T2DM, Proteinuria, Therapeutic inertia, Primary care, Risk factors

## Abstract

**Background:**

Therapeutic inertia (TI), defined as physicians’ failure to increase therapy when treatment goals are unmet, is an impediment to chronic disease management. This study aimed to identify the prevalence of TI in proteinuria management among T2DM patients managed in primary care settings and to explore possible associating factors.

**Methods:**

This was a cross-sectional study. T2DM patients with proteinuria (either microalbuminuria or macroalbuminuria) and had been followed up in 7 public primary care clinics of the Hospital Authority of Hong Kong from 1 Jan, 2014 to 31 Dec, 2015 were included. The prevalence of TI in proteinuria management and its association with patients’ demographic and clinical parameters and the working profile of the attending doctors were explored. Student’s t test and analysis of variance were used for analyzing continuous variables and Chi square test was used for categorical data. Multivariate stepwise logistic regression was used to determine the association between TI and the significant variables from patients' and doctors' characteristics.

**Results:**

Among the 22,644 T2DM patients identified in the case register, 5163 (26.4%) patients were found to have proteinuria. Among the sampled 385 T2DM patients with proteinuria, TI was identified in 155 cases, with a prevalence rate of 40.3%. Male doctor, doctor with longer duration of clinical practice and have never received any form of Family Medicine training were found to have a higher TI. Patients with microalbuminuria range and lower systolic and diastolic blood pressure (BP) were also found to have higher TI. Logistic regression study revealed that patients’ systolic BP level and microalbuminuria range of proteinuria were negatively associated with the presence of TI, whereas doctor’s year of clinical practice being over 20 years and patients being treated with submaximal dose of medication were positively associated with the presence of TI.

**Conclusions:**

TI is commonly present in proteinuria management among T2DM patients, with a prevalence of 40.3% in primary care. Systolic BP and microalbuminuria range of urine ACR were negatively associated with the presence of TI, whereas submaximal ACEI/ARB dose and doctors practicing over 20 years were positively associated with the presence of TI. Further studies exploring the strategies to combat TI are needed to improve the clinical outcome of T2DM patients.

## Background

Type 2 diabetes mellitus (T2DM) affects about 10% of the Hong Kong population and is a leading cause of morbidity and mortality due to its complications [1]. Among all diabetic complications, the presence of proteinuria including microalbuminuria and macroalbuminuria is a well-known predictor of poor renal and cardiovascular outcomes [2, 3]. Proper management of proteinuria has been proven to reduce the risk of adverse renal and cardiovascular events [4, 5].

Effective treatments to prevent the development and progression of diabetic nephropathy (DMN) are available. For example, angiotensin-converting enzyme inhibitors (ACEI) reduced the onset of microalbuminuria and macroalbuminuria in T2DM patient with or without hypertension [6, 7]. Meta-analysis also revealed similar beneficial benefits from Angiotensin II receptor blockers (ARB) in high risk T2DM patients [8, 9]. Based on these evidence, the American Diabetes Association (ADA) guidelines on T2DM management since 2013 recommends that all T2DM patients with micro- or macroalbuminuria should be treated with an ACEI or ARB to mitigate the progression of DMN [10].

Despite all these evidence, proteinuria control among diabetic patients has been inadequate in primary care setting both internationally and locally. For example, studies from the United States have shown that the overall prevalence of micro-, and macroalbuminuria was 39% and 10% respectively among T2DM patients [11]. However, only 25% of them were treated with ACEI or ARB [12]. Similar studies carried out at local primary care settings revealed that the prevalence of microalbuminuria among T2DM patients was 13.4% [13] and about half of T2DM patients with proteinuria were treated with ACEI or ARB [14].

Reasons of the suboptimal proteinuria control among T2DM patients are multifactorial, which might include patient, physician and system factors. Among them, therapeutic inertia (TI), defined as physicians’ failure to initiate or increase therapy when treatment goals are unmet, has been identified as an important physician factor. Indeed, more and more evidence has shown that failure of health care providers to initiate or intensify therapy according to updated guidelines is an impediment to chronic disease management [15]. For example, TI is commonly observed in diabetes and hypertension management and has led to a significantly higher rate of heart attack and stroke [16, 17].

Locally, a significant proportion of T2DM patients are managed in the primary care and are followed up at government public clinics under the Hospital Authority of Hong Kong (HAHK). Up to now, there was no study exploring the prevalence of TI in proteinuria management among T2DM patients both internationally and locally. To fill this knowledge gap, this study aimed to explore the prevalence of TI in proteinuria management among T2DM patients and to explore possible associating factors from both patients’ and doctors’ perspective. It is hoped that, by early identifying the risk factors for TI and taking proactive measures to combat the TI, proteinuria management could be enhanced in primary care settings and therefore the renal and cardiovascular outcome of T2DM patients could be improved in the long run.

## Method

### Study design

Cross-sectional study.

### Subjects

T2DM patients coded with International Classification of Primary Care-2 (ICPC-2) T90 (Non-insulin Dependent Diabetes Mellitus), who had attended General Outpatient Clinic (GOPCs) of Kowloon Central Cluster (KCC) of HAHK from 1 Jan 2014 to 31 Dec 2015 for disease management, and had diabetes blood and urine test at least once during this period were included. Diabetic complication screening would be performed for all T2DM patients in our clinics at every 1 to 2 years’ interval. Totally there are 7 clusters in HAHK, with KCC being one of the hospital cluster with a catchment of 0.5 million populations in 2015. There are 7 GOPCs under the jurisdiction of KCC, and around 15% of the population were aged over 65 years in 2014–2015. The diagnosis of diabetes was based on the “Definition and description of diabetes mellitus” from American Diabetes Association in 2013 [10]. Type 1 diabetes patients, T2DM patient being FU at specialist setting, or T2DM patients without any blood or urine check-up during the study period were excluded.

### Definition of proteinuria (microalbuminuria and macroalbuminuria) among T2DM patients

According to ADA guideline 2013, all T2DM patients should have urine screening for microalbuminuria [10]. Microalbuminuria was defined as albumin secretion of 30 to 300 mg/24 h and macroalbuminuria or overt proteinuria as a value of > 300 mg/24 h. Screening for microalbuminuria can be performed by measurement of urine albumin over creatinine ratio (ACR) in a random spot collection. Normo-albuminuria was defined as a urine ACR of < 2.5 mg/mmol in males and < 3.5 mg/mmol in females. Corresponding values for microalbuminuria were defined as 2.5 to 30 mg/mmol for males and 3.5 to 30 mg/mmol for females. For macroalbuminuria, the urine ACR is > 30 mg/mmol for both genders. An elevated urine ACR needed to be confirmed in the absence of urinary tract infection with additional first-void specimens collected during the next 3 to 6 months [18].

### Definition of therapeutic inertia in management of proteinuria among T2DM patients

In this study, management of proteinuria is considered as inadequate and escalation of treatment is indicated if the latest urine ACR level is ≥ 2.5 mmol/L in male patients and ≥ 3.5 mmol/L in female patients. Consultation notes immediately after the latest urine ACR test being available were reviewed through the computer management system (CMS). TI is considered to be present when the attending doctors failed to initiate or intensify ACEI/ARB treatment to address the proteinuria control. On the other hand, if there are valid reasons documented in the medical notes justifying that initiating or escalating treatment is not feasible despite clinical indications, it will not be counted as TI.

Common justifications included:

1. ACEI/ARB is contraindicated due to drug allergy, with history of renal artery stenosis, or patients’ blood pressure on attendance has been low (< 90/60mmhg);
2. Patients’ intolerance to the side effects of ACEI/ARB, such as with prior history of drug induced renal impairment or hyperkalemia (serum potassium > 5.0 mmol/L) etc. As patients who could not tolerate ACEI due to persistent cough could be treated by ARB, failing to transfer the treatment regime will also be counted as TI.
3. Other causes of increased ACR need to excluded, such as urinary tract infection etc.
4. Patients’ non-compliance to the existing ACEI/ARB regime and advice on regular drug compliance was given;
5. Patients’ refusal to start ACEI/ARB.
6. Patients are taking the maximum dosage of ACEI/ARB

### Sample size calculation and random sampling

According to the baseline data from Clinical Data Analysis and Reporting System (CDARS) of HAHK, totally 22,644 DM patients had been managed in 7 GOPCs of KCC. Among them, 19,583 patients had diabetes blood and urine ACR checked at least once during the study period. Based on our definitions mentioned above, 5,163 of them had diabetic nephropathy with either micro- or macroalbuminuria. By using the sample size calculator from Creative Research System (http://www.surveysystem.com), 385 cases would be needed to provide 95% confidence level and 5% confidence interval. To ensure adequate statistical power and to allow room for case exclusion, a roundup of 400 cases were included for the study.

A list of 400 random numbers were generated from the research randomizer (http://www.randomizer.org/form.htm), from which the 400 sampled T2DM patients were selected. Details of the clinical consultation when the elevated urine ACR result had been available were reviewed. Data were collected by using a standard data collection form by the principle investigator and counter checked by another experienced doctor in the research team.

### Determination of variables

The recruited patients’ demographic data, latest blood pressure (BP), Hemoglobin A1c (HbA1c), serum creatinine level, lipid profile and urine ACR were retrieved from the CMS. If more than one test had been performed during the study period, the most recent blood or urine test would be used for data analysis. Abbreviated Modification of Diet in Renal Disease (MDRD) formula was used to calculate the estimated Glomerular Filtration Rate (eGFR).

The working profiles of the attending doctors were provided by the Central Administration Office of Department of Family Medicine (FM) and GOPC, KCC. Duration of clinical practice was calculated as the number of years after registration in the Medical Council of Hong Kong. Training status of attending doctors was categorized according to the following criteria:

- Group 1: Service doctors without FM training before.
- Group 2: Trainee doctors undergoing basic vocational training from Hong Kong College of Family Physicians (HKCFP), or studying the Diploma of Family Medicine (DFM).
- Group 3: Intermediate fellow doctors who had obtained the conjoint fellowship in HKCFP.
- Group 4: FM specialists who are fellow of Hong Kong Academy of Medicine (HKAM), specialized in FM.

### Statistical analysis

All data were entered and analyzed using computer software SPSS version 21.0 (SPSS Inc, Chicago [IL], US). Chi square test were used to analyze the categorical data and Student’s t test were used for continuous variables. Fisher’s test was used if the sample size was less than five. The association between TI and patients' and doctors' characteristics were explored by multivariate stepwise logistic regression analysis. All statistical tests were two sided, *P* < 0.05 was considered to be statistically significant.

### Ethical consideration

The Ethical Approval was granted by the Research Ethical Committee (Kowloon Central/Kowloon East Cluster) on 5/8/2016, the reference number is KC/KE-16–0109/ER-1.

## Results

**Fig. 1:**
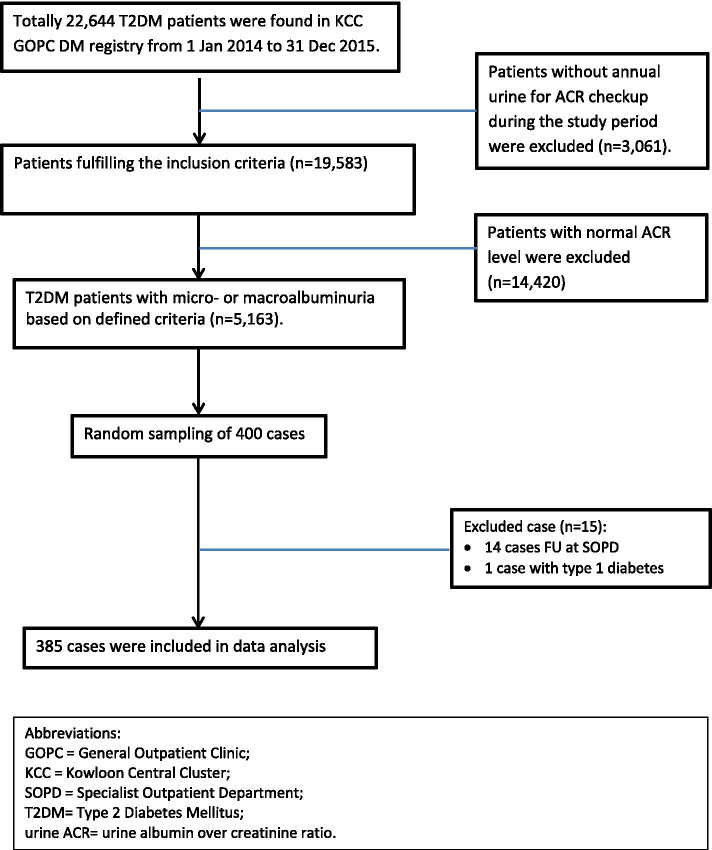
Flow chart of patient recruitment of this study

A total of 22,644 T2DM patients were identified from the T2DM registry from 1 Jan, 2014 to 31 Dec, 2015. Among them, 19,583 (86.5%) patients had their blood and urine ACR checked at least once during the above period; 5,163 (26.4%) patients were found to have DMN with microalbuminuria (*n* = 4,132, prevalence rate 21.1%) or macroalbuminuria (*n* = 1,031, prevalence rate 5.3%). Among the 400 randomly sampled DMN patients, 15 cases were excluded including 14 cases being FU by Specialist setting and 1 case with type 1 diabetes. The remaining 385 patients were included for data analysis (Fig.1).

Table 1 summarized the demographic characteristics of the recruited patients. The mean age of the study population was 70.0 ± 12.6 years and 195 patients (50.6%) were female. The mean duration of diabetes was 9.8 ± 8.1 years. With regard to their co-morbidities, 360 (93.5%) patients had concomitant hypertension (HT), 20 (5.2%) had ischemic heart disease (IHD), 43 (11.2%) had cerebral vascular accident (CVA), and 1 (0.3%) had peripheral vascular disease (PVD). Mean urine ACR level was 35.9 ± 84.0 mg/mmol. 292 (75.8%) had microalbuminuria and 93 (24.2%) had macroalbuminuria. Among them, half were treated with ACEI (*n* = 194) and 16.1% were treated with ARB (*n* = 62).

**Table 1:** Demographic and clinical characteristics of T2DM patients included in the study

Patient characteristics	Total number (*n* = 385)
Sex	
Male	190 (49.4%)
Female	195 (50.6%)
Age (years)	70.03 ± 12.58
BMI (kg/m^2^)	25.95 ± 4.75
Smoking	
Non-smoker	274 (71.2%)
Ex-smoker	72 (18.7%)
Smoker	39 (10.1%)
Duration of DM (years)	9.76 ± 8.05
Hemoglobin A1c (%)	6.87 ± 1.17
eGFR (mL/min/1.73m^2^)	70.45 ± 24.36
Blood Pressure	
Systolic BP (mmHg)	131.9 ± 17.0
Diastolic BP (mmHg)	71.4 ± 11.7
urine ACR level (mg/mmol)	35.9 ± 84.0
Co-morbidities:	
HT	360 (93.5%)
CVD (IHD or CVA or PVD)	
IHD	20(5.2%)
CVA	43 (11.2%)
PVD	1 (0.3%)
Lipid Profile	
TG (mmol/L)	1.52 ± 1.1
TC (mmol/L)	4.28 ± 0.91
HDL (mmol/L)	1.32 ± 0.38
LDL (mmol/L)	2.27 ± 0.75
Current use of ACEI/ARB	
ACEI	194 (50.4%)
ARB	62 (16.1%)

Data are shown as mean ± standard deviation or No. (%) of cases

*Abbreviations*: *BMI*  Body mass index, *DM* Diabetes mellitus, *eGFR* Estimated glomerular filtration rate, *BP* Blood pressure, *ACR* Albumin over creatinine ratio, *HT* Hypertension, *CVD* Cardiovascular disease, *IHD* Ischaemic heart disease, *CVA* Cerebrovascular, *PVD* Peripheral vascular disease, *TG* Triglyceride, *TC*  Total cholesterol, *HDL* High-density lipoprotein, *LDL*  Low-density lipoprotein, *ACEI* Angiotensin converting enzyme inhibitor, *ARB* Angiotensin receptor blocker

Table 2 summarized the demographic profiles of physicians who have attended the recruited T2DM cases with proteinuria. A total of 58 doctors, among whom 23 (39.7%) were female, attended the 385 T2DM patients. The mean duration of clinical practice was 15.4 ± 9.2 years. With regard to FM training status, 13 (22.4%) doctors had received no FM training, 13 (22.4%) received basic training, 16 (27.6%) were intermediate FM fellows, and 16 (27.6%) were FM specialists. Sub-analysis of attending doctors’ profile according to their duration of clinical practice and FM training status is shown in Table 3. Training status of FM varied significantly with duration of clinical practice (*P* < 0.001). Among 7 doctors who had worked for ≤ 5 years, all had been receiving basic FM training or had become intermediate fellow. On the other hand, among 15 doctors who had worked for over 20 years, most (*n* = 12, 80%) had not received any formal FM training.

**Table 2:** Demographic and training profile of physicians who have attended the recruited T2DM cases with proteinuria

Physicians characteristics	Total number (*n* = 58)
Sex	
Male	35
Female	23
Duration of clinical practice (years)	15.4 ± 9.2
Duration of clinical practice (years)	
≤ 5	7
6–10	14
11–19	22
≥ 20	15
FM training status	
None	13
Basic FM trainee/DFM	13
Intermediate fellow	16
HKAM FM specialist	16

Data are shown as mean ± standard deviation or number (n) of cases

**Table 3:** Sub-analysis of attending doctors’ profile according to duration of clinical practice and FM training status

Duration of clinical practice	FM training status					*P* value
	None	Basic FM trainee/DFM	Intermediate fellow	HKAM FM specialist	Total	
** ≤ 5 years**	0	6	1	0	7	**0.000**
**6–10 years**	0	5	8	1	14	
**11–19 years**	1	2	6	13	22	
** ≥ 20 years**	12	0	1	2	15	
**Total**	13	13	16	16	58	

*Abbreviations*: *FM* Family Medicine, *DFM* Diploma in Family Medicine, *HKAM* Hong Kong Academy of Medicine

Table 4 shows the characteristics of physicians in TI-positive and TI-negative patients. Among 385 T2DM with micro- or macroalbuminuria, TI was identified in 155 cases, with a prevalence rate of 40.3%. Male doctors were found to have a higher TI rate (45.9%) compared with female doctors (31.6%, *P* = 0.006). The duration of clinical practice of attending doctors was significantly longer in the TI group (17.6 ± 9.4 yrs) compared with non-TI group (13.8 ± 7.3 yrs, *P* < 0.001), with doctors working for over 21 years having a particularly higher rate of TI (52.7%). With regards to training status, doctors without any form of FM training were found to have a much higher rate of TI (54.7%, *p* = 0.002). However, among the subgroups of doctors with different level of training, i.e. basic trainee, intermediate fellow or FM specialist, no significant difference was identified (*p* = 0.313).

**Table 4:** Comparisons of the prevalence of TI according to attending doctors’ profile

Doctor’s profile	Total cases (*n* = 385)	With TI (*n* = 155)	Without TI (*n* = 230)	Prevalence of TI = 40.3%	*P* value
Sex					**0.006**
Male	233	107	126	45.9%	
Female	152	48	104	31.6%	
Duration of clinical practice					
Average (years)	15.3 ± 8.4	17.6 ± 9.4	13.8 ± 7.3		**0.000**
≤ 5 yrs	24	9	15	37.5%	**0.005**
6–10 yrs	119	34	85	28.6%	
11–19 yrs	151	64	87	42.4%	
≥ 20 yrs	91	48	43	52.7%	
Training status					
Non	86	47	38	54.7%	**0.002**
With FM training	299	108	191	36.1%	**0.313**
Basic FM trainee/DFM	74	26	48	35.1%	
Intermediate fellow	130	42	88	32.3%	
HKAM FM specialist	95	40	55	42.1%	

Data are shown as mean ± standard deviation or No. (%) of cases

Table 5 summarized the characteristics of T2DM patients in TI-positive and TI-negative groups.

**Table 5:** Study on patients’ profile in the presence or absence of therapeutic inertia (TI) *

Patients characteristics	Without TI (*n* = 230)	With TI (*n* = 155)	*P*-value
Sex			0.604
Male	116	74	
Female	114	81	
Age (years)	69.6 ± 11.8	70.6 ± 13.7	0.471
BMI (kg/m^2^)	25.37 ± 4.85	25.33 ± 4.53	0.603
Smoking			0.543
Never smoke	168	106	
Ex-smoker	39	33	
Smoker smoke	23	16	
Duration of DM (years)	9.46 ± 7.5	10.2 ± 8.8	0.366
Haemoglobin A1c (%)	6.88 ± 1.22	6.85 ± 1.11	0.811
eGFR (ml/min/1.73m^2^)	70.1 ± 24.4	71.0 ± 24.4	0.702
Urine ACR level (mg/mmol)	39.1 ± 72.4	31.0 ± 98.8	0.356
2.5/3.5–30	160 (69.6%)	132 (85.2%)	0.001
30–300	66 (28.7%)	20 (12.9%)	
> 300	4 (1.7%)	3 (1.9%)	
Blood pressure (mmhg)			
Systolic	134.6 ± 18.6	128.0 ± 13.6	0.000
Diastolic	72.5 ± 12.2	69.9 ± 10.8	0.036
Treatment with ACEI/ARB			
Nil	64 (28.8%)	64 (41.3%)	0.000
On submaximal dose of ACEI/ARB	46 (20.0%)	88 (56.8%)	
On maximal dose of ACEI/ARB	120 (52.2%)	3 (1.9%)	
Co-morbidities:			
HT	220 (95.7%)	140 (90.3%)	0.037
CVD			
IHD	11 (4.8%)	9 (5.8%)	0.657
CVA	30 (13.1%)	15 (9.7%)	0.371
PVD	0 (0%)	1 (0.6%)	0.403
Other diabetic complications			
Diabetic retinopathy	98(42.6%)	65 (41.9%)	0.90
Diabetic neuropathy	36 (15.7%)	21 (13.5%)	0.569
Lipid Profile			
TG (mmol/L)	1.50 ± 1.05	1.55 ± 1.18	0.654
TC (mmol/L)	4.31 ± 0.9	4.23 ± 0.93	0.429
HDL (mmol/L)	1.34 ± 0.39	1.29 ± 0.37	0.203
LDL (mmol/L)	2.29 ± 0.71	2.25±0.80	0.553

Data are shown as mean ± standard deviation or No. (%) of cases

*Abbreviations*: *BMI*  Body mass index, *DM* Diabetes mellitus, *ACR* Albumin over creatinine ratio, *eGFR* Estimated glomerular filtration rate, *ACEI*  Angiotensin converting enzyme inhibitor, *ARB* Angiotensin receptor blocker, *HT* Hypertension, *CVD* Cardiovascular disease, *IHD* Ischaemic heart disease, *CVA* Cerebrovascular accident, *PVD* Peripheral vascular disease, *TG* Triglyceride, *TC * Total cholesterol, *HDL*  High-density lipoprotein, *LDL* Low-density lipoprotein

Patients from both groups were comparable in terms of male to female ratio, age, BMI, duration of DM, Hemoglobin A1c level, eGFR level and urine ACR level. In addition, their complication rate with stroke, IHD, PVD and diabetic complications including diabetic retinopathy and diabetic neuropathy were also similar (all *P* > 0.05). However, most of TI-positive DM patients (85.2%) were in the micro-albuminuria range versus 69.6% in TI-negative group (*P* = 0.001). The average systolic and diastolic BP was also found to be lower in TI-positive group than TI-negative group (*P* = 0.000 and *P* = 0.036).

Based on the results from Tables 4 and 5, multivariate stepwise logistic regression analysis was performed to identify any factors that contributed to TI (Table 6). Only variables that were significantly different in the univariate analysis were included in the regression model.

**Table 6:** Logistic regression analysis on associating factors contributing to the presence of therapeutic inertia in proteinuria management among T2DM patients

Independent variables	Odds ratio	95% C.I.for EXP(B)		*P* value
		Lower	Upper	
Systolic blood pressure reading	0.97	0.95	0.99	0.001
Urine ACR being 2.5/3.5 to 30 mg/mmol	0.37	0.18	0.73	0.004
On submaximal dose of ACEI/ARB	2.40	1.30	4.44	0.005
Drs’ year of practice (≥ 20 yrs)	4.29	1.64	11.19	0.003

 As the FM training status varied significantly with the duration of clinical practice and these two factors were interrelated (Table 3, *P* < 0.001), only one of these two variables was included in the logistic regression analysis. As the *P* value of years of clinical practice (*P* < 0.000) was smaller than that for FM training status (*P* = 0.006) in the univariate analysis (Table 4), only doctors’ year of clinical practice was entered into the logistic regression analysis. Logistic regression study revealed that the systolic BP reading and microalbuminuria range of proteinuria were negatively associated with the presence of TI, whereas doctors’ year of clinical practice being over 21 years and patients being treated with submaximal dose of ACEI/ARB were positively associated with the presence of TI.

## Discussion

Microalbuminuria is the earliest sign of DMN and predicts increased cardiovascular mortality and morbidity and end stage renal failure. Our study revealed that the prevalence of microalbuminuria and macroalbuminuria among T2DM patients from primary care was 21.1% and 5.3% respectively. These figures are higher than those reported from similar studies done in a primary civil servant clinic in HK which showed that the prevalence of microalbuminuria was 13.4% among T2DM patients [13]. It is noted that the average age of patients in their study was 58 years old, which is much younger than cases recruited in this study (70 years). Since advanced age is an independent risk for the development of microalbuminuria, a higher prevalence rate of microalbuminuria in our study is expected. In addition, multiple studies have demonstrated that the prevalence of microalbuminuria varies among races, even within the same community [19]. For example, cross-sectional studies have reported marked variation in the prevalence of microalbuminuria, ranging from 14.2% in Singapore [20], around 30% in the United States [11] to over 40% in China [21]. These variations in prevalence can be attributed to differences in the definition of microalbuminuria, method of urine collection and different ethnic susceptibility to nephropathy.

Among T2DM patients included in the data analysis, 360 cases (93.5%) were found to have concomitant HT, whereas only 66.5% of them were treated with ACEI or ARB. It is consistent with findings from another local study from primary care clinics showing that only about half of diabetic patients with micro- or macroalbuminuria were treated with ACEI or ARB [14]. Based on our set criteria, TI was found present in 40.3% cases, meaning that in over 40% of T2DM patients with proteinuria, ACEI or ARB initiation or dose augmentation had not been provided. This high TI rate may contribute to the suboptimal proteinuria control among T2DM patients managed in primary care. As there are no similar studies carried out internationally or locally on this particular topic, direct comparisons with other studies are not possible. Having said so, this TI rate in proteinuria management was much higher than the TI rate in glycaemic control (29–33%) among T2DM patients [22, 23], although lower than the TI rate in blood pressure (63.3–68%) [24] and lipid control (66–80%) [25] carried out by various research teams. This relatively high TI rate should alert family physicians the importance of proteinuria control among T2DM patients and make concerted effort to combat the TI.

Further studies on the physician’s profile revealed that male doctors, doctors with longer duration of clinical practice, particularly those with over 20 years’ clinical practice, and doctors without FM training had a higher rate of TI. In our study, most doctors who had worked for over 20 years (80%) had not received any formal FM training before. In addition, doctors with FM training had a much lower rate of TI than those without (36.8% vs 53.8%, *P* = 0.006). Therefore, we postulate that doctors who have worked for over 20 years had a higher inertia rate possibly due to a lack of FM or related training. If physicians lack appropriate training, there will be knowledge gaps and subsequently suboptimal care. Indeed, some evidence suggests that physicians who have been in practice for more years may be less likely to deliver high-quality care or comply with treatment guideline [26, 27]. Medical advances occur frequently, and the explicit knowledge that physicians possess may easily become out of date. Therefore, although it is generally assumed that the knowledge and skills accumulated by physicians during years of practice lead to superior clinical abilities; it is plausible that physicians with more experience may paradoxically be less likely to provide technically appropriate care. This has been confirmed by a systematic review which showed that, among 62 published studies that measured physician knowledge or quality of care and the time since medical school graduation or age, more than half suggested that physician performance declined over time for all outcomes measured [28]. Therefore, these findings raise concerns about the adequacy of continuing professional education in medicine and alert us the need to provide quality improvement interventions to this subgroup of doctors.

With regards to patient’s profile, we found that TI was more prominent in patients with microalbuminuria (85.2% of all TI patients). This could be explained by the threshold effect which is, the closer the urine ACR level is to normal level, the less likely the doctor to intensify the treatment. Indeed, the threshold effect has been observed in other conditions such as glycemic and lipid management in T2DM patients [22, 25]. Besides, “complacency with borderline values” may also lead to the physician’s subjective misperception that the care provided is sufficient. In addition, we found that T2DM patient in the TI group had a lower systolic and diastolic BP compared with non-TI group. 65.8% of TI group has satisfactory BP control (< 130/80mmhg) at the clinical visit compared with non-TI group (47.8%, *P* = 0.0005). These data revealed that doctors are less likely to initiate or intensify ACEI or ARB treatment in normotensive albuminuria cases despite the evidence that ACEIs and ARBs reduce the risk of progression to macroalbuminuria in normotensive T2DM patients with microalbuminuria. Possible reasons might include concerns about the development of hypotension if ACEI/ARB is initiated or the dose is augmented. The side effects of ACEI or ARB, such as hyperkalemia and persistent throat discomfort and dry cough, are other common concerns. Although these concerns sound reasonable, lots of studies have proven that albuminuria control slows the progression of DMN and improves the clinical outcomes among T2DM patients even in the absence of HT [29]. In line with these findings, international guidelines recommended that ACEIs or ARBs should be initiated in T2DM cases with micro-albuminuria unless contraindicated [30]. Therefore, clinicians should be more proactive in initiating proteinuria control treatment to prevent the disease progression. At the same time, doctors should also strike a balance between the benefit of proteinuria control and the possible risk of hypotension and closely monitor the BP reading during FU consultations.

This study is the first clinical analysis of TI in proteinuria management among T2DM patients internationally. It has added new knowledge on the prevalence of TI in proteinuria management among diabetic patients and explored the possible underlying factors from both the doctor’s and patient’s perspective. These findings will help inform policy makers about future directions of strategic planning to combat the TI and therefore enhance the proteinuria management among T2DM patients. As all data on proteinuria, comorbidities and the drug profile were retrieved from the CMS of HKHA, data entry bias or recall bias were minimized. That being said, limitations do exist for this study. First, the study was carried out in one single cluster of HAHK and therefore selection bias might exist. These results from the public primary health care sector might not be applicable to the private sector or secondary care. Second, T2DM patients who had not checked blood or urine test during the study period were excluded (*n* = 3,061, 13.5% of all diabetic cases). The urine ACR status of this group of diabetic patients remained unknown. This might bias the accurate measurement of TI among our target population. Lastly, as this study relied heavily on review of consultation notes to identify the justifications of not initiating or augmenting ACEI or ARB, insufficient documentation may have resulted in an overestimation of the prevalence of TI.

Considering that large numbers of T2DM patients are managed in the primary care setting and the importance of proteinuria control, comprehensive strategies to combat TI in proteinuria management should be implemented to improve the disease outcome. Structured continuous medical education and professional training to primary care doctors on chronic disease management should be intensified to mitigate the TI. Future interventional studies are also needed to evaluate the effectiveness of different improvement strategies, both on patients’ and doctors’ perspective, in combating the TI.

## Conclusion

Therapeutic inertia is commonly seen in the management of proteinuria among T2DM patients, with a prevalence of 40.3% in primary care. Systolic BP and microalbuminuria range of urine ACR were negatively associated with the presence of TI, whereas submaximal ACEI/ARB dose and doctors practicing over 20 years were positively associated with the presence of TI. Comprehensive strategies should be devised to overcome TI so as to enhance proteinuria management among T2DM patients and subsequently improve their renal and cardiovascular outcome.

## Abbreviations

ACEI: Angiotensin-converting enzyme inhibitor
ACR: Albumin over creatinine ratio
ADA guideline: American Diabetes Association guideline
ARB: Angiotensin II receptor blocker
BMI: Body mass index
BP: Blood pressure
CDARS: Clinical Data Analysis and Reporting System
CMS: Clinical Management System
CVA: Cerebral vascular accident
CKD: Chronic kidney disease
DMN: Diabetic Nephropathy
eGFR: Estimated Glomerular Filtration Rate
FU: Follow Up
FM: Family Medicine
GOPCs: General Outpatient Clinics
HAHK: Hospital Authority of Hong Kong
HbA1c: Hemoglobin A1c
HK: Hong Kong
HT: Hypertension
HKCFP: Hong Kong College of Family Physician
HKAM: Hong Kong Academy of Medicine
ICPC: International Classification of Primary Care
IHD: Ischemic Heart disease
KCC: Kowloon Central Cluster
PVD: Peripheral vascular disease
SOPCs: Specialist Outpatient Clinics
T2DM: Type 2 diabetes mellitus
TI: Therapeutic inertia
